# Resilience and self-harm among left-behind children in Yunnan, China: a community-based survey

**DOI:** 10.1186/s12889-019-8075-4

**Published:** 2019-12-23

**Authors:** Xin Tian, Wei Chang, Qiong Meng, Ying Chen, Zhen Yu, Limei He, Yuanyuan Xiao

**Affiliations:** 1 Department of Epidemiology and Health Statistics, School of Public Health, Kunming Medical University, Kunming, 650500 Yunnan China; 2 The First Affiliated School of Clinical Medicine, Kunming Medical University, Kunming, Yunnan China; 3 Department of Chronic Disease Epidemiology, Yale School of Public Health Yale University, New Haven, CT USA

**Keywords:** Left-behind children, Self-harm, Resilience, Emotion regulation, Family support

## Abstract

**Background:**

Self-harm (SH) behaviors are established risk factors of suicide, however, in Chinese left-behind children (LBC), SH remains severely under-discussed. In this study, we aimed to investigate SH and explore its association between resilience in a large group of LBC.

**Methods:**

A community-based cross-sectional study of 2898 LBC was conducted in Yunnan province, China. Information was collected by self-reporting questionnaires. Descriptive analysis was used to depict and compare characteristics of the subjects. Univariate and multivariate logistic regression models were applied to estimate the associations between resilience and SH prevalence, SH severity, SH repetition.

**Results:**

The prevalence of SH in LBC was 48.8% (95%CI: 47.0–49.7%). Compared to LBC with lower level of resilience, a higher level of resilience was related to 0.40 folds of SH odds (95%CI: 0.34–0.48). Besides, among all dimensions of resilience, every 1 average score increase of emotion regulation and family support were associated with 0.13 (95%CI: 0.04–0.37) and 0.14 (95%CI: 0.04–0.47) folds of odds in severer SH, respectively; one unit increase in the average score of emotion regulation was related to an OR of 0.23 (95%CI: 0.07–0.71) for repeated SH.

**Conclusions:**

Psychological resilience presented protective effect on SH in LBC, especially the dimensions of emotion regulation and family support. Intervention measures focusing on enhancing emotion regulation ability and building parent-child connection could be considered in order to reduce SH and suicidal risk in LBC.

## Background

With the widening rural-urban split exacerbated by economical prosperity and urbanization in the last four decades in China, increasing flow of rural labors into big cities for job opportunities has generated an expanding population known as the left-behind children (LBC), which indicates to children been left at home by one or both of their migrated parents [1, 2]. According to the released data in 2013 by All China Women’s Federation, the number of LBC had climbed to a staggering 61 million, accounted for 37.7% of the rural children and 21.9% of the total Chinese children [3, 4]. The overwhelmingly majority of LBC are from inland destitute areas, which made LBC a socioeconomically deprived population. Moreover, compared to non-left-behind children (NLBC), the long separation from parents will put LBC into an even more vulnerable situation. Therefore, in recent years, concerns regarding to the health status of LBC have been accumulated [5–7].

Self-harm (SH) refers to the behaviors that individual adopted to hurt oneself regardless of the intention [8]. It can occur at any age but reportedly more common in pubertal phase [9]. In China, a multi-center study based on a sample of 11,880 adolescents reported a SH prevalence of 30.3% [10]. Individual, familial, and social environmental determinants may collectively contribute to the increased SH in adolescents, and among them, it has been suggested that living in structurally or functionally abnormal family was associated with increased risk of SH [11]. Therefore, it is reasonable to suspect that, SH incidence of LBC will be significantly higher than NLBC. In fact, two previous cross-sectional studies published in Chinese have preliminarily investigated this issue with supportive evidence [12, 13]. Existing studies highlight the importance of SH in suicide, as it has been estimated that approximately 50–60% of suicidal deaths were coupled with a history of SH, and SH adolescents reported threefold risk of suicidal ideation (SI) compared with their non-SH counterparts [14, 15]. A recently published study found that, the one-year prevalence of suicide attempt in Chinese LBC was 3.75%, significantly higher than which in NLBC (2.86%) [16]. Therefore, it is of great importance to investigate SH and its influencing factors in Chinese LBC, so as to effectively reduce future suicidal risk in this vulnerable population. However, up till now, this topic remains severely under-discussed.

In the field of psychology, resilience is described as a competence of individual to successfully pull through adversity or trauma [17]. A newly published meta-analysis revealed a lower resilience level of Chinese LBC than NLBC [18]. Western literature reported that resilience was significantly associated with SH: SH individuals were generally observed lower level of resilience [19]. With this regard, resilience building might be a promising method to alleviate SH frequency and severity among LBC. It is also practical, for empirical evidence has suggested that individual resilience can be drastically reinforced by psychological or psychosocial intervention measures [20]. Nevertheless, this possible protective role of resilience in SH has never been explored in Chinese LBC.

Aiming at the deficiencies in current literature mentioned above, we intended to intensively analyze the association between resilience and SH in a large community-based sample of Chinese LBC. We hypothesized that, resilience is inversely associated with SH prevalence, SH severity, and SH repetition.

## Methods

### Study population and procedure

After the Ethics Review Board of Kunming Medical University approved, we performed a cross-sectional survey in Guangnan, a county affiliated to Wenshan Zhuang and Miao Autonomous Prefecture, home to the largest number of LBC among ethnical minority prefectures in Yunnan province, China. The survey was performed from June 26 to July 6, 2018. A one-stage random cluster sampling was applied: 1) Three townships were randomly chosen from a total of 18 rural townships in Guangnan; 2) All eligible LBC within the chosen townships were preliminarily included. We then used the following inclusion criteria to further screen study subjects: 1) One or both of their parents had been migrated for at least 6 months in the most recent year; 2) Aged between 10 and 17 years; 3) Resided in survey sites for at least six months per year. Children or adolescents were further excluded if they were: 1) Illiterate; 2) Delirious or suffering from severe psychiatric disorder with incompetence in expression; 3) Hearing or speech dysfunction; 4) Critically ill or under inconvenient conditions; 5) Refuse to participate.

*Sample size.* We chose a conservative estimation of 20% for SH prevalence based on published literature. The acceptable error rate and statistical significance level were set as 3% and 0.05, respectively. By using the formula for simple random sampling ($N=\frac{{Z^2}_{2/\alpha}\pi \left(1-\pi \right)}{\delta^2}$), we calculated a preliminary sample size of 712. Given the possible advent of refusal, an adjusted sample size was obtained on the premise of 80% effective response rate (*N* = 712/0.8 = 890). Considering that the sampling error of cluster sampling is inevitably bigger that simple random sampling, a design effect of 2 was applied to further adjust the required sample size, therefore, the final calculated sample size was *N* = 890 × 2 = 1780.

*Measurements.* Face-to-face interview was applied to collect information from study subjects by paper-and-pencil approach. Interviewers were qualified senior class undergraduates or graduates majoring in clinical medicine or public health from Kunming medical university. Before survey, all interviewers accepted a one-day intensive training and passed the subsequent examination. Prior to interview, a written consent was obtained from either the parent or legal guardian or teacher of the participant, as appropriate to the specific situation. The instrument we used contains 5 parts, which measure general characteristics, SH behaviors, psychological resilience, depressive symptoms (measured by the Chinese Version of Children’s Depression Inventory), and SI (measured by the Chinese Version of Beck Scale for Suicide Ideation) of LBC, respectively. The current study only used variables from the first three parts.

*Self-harm.* Self-harm was measured by using the Modified version of Adolescents Self-Harm Scale (MASHS) developed by Feng [21]. MASHS measures both the lifetime frequency and severity of the 18 most commonly reported SH behaviors among Chinese adolescents. Frequency of SH was measured by scaled options: never, once, two to four times, five times and above. SH severity was divided into 5 levels: non-observable injury, slight injury (observable but no need to treat), medium injury (requires simple treatment, no need to visit medical facilities), severe injury (requires treatment in medical facilities, no need to be hospitalized), critical injury (requires urgent treatment in emergency room and the subsequent hospitalization). The Cronbach’s α of MASHS in this sample was 0.75, indicates an acceptable level of internal consistency.

*Resilience.* We adopted the Resilience Scale for Chinese Adolescents (RSCA) designed by Hu and Gan [22]. The respondents were expected to answer the degree of pertinence of the 27 items based on their own situation. Answers were rated by a five-point Likert scale: totally disagree (1 point), disagree (2 points), not sure (3 points), agree (4 points), and totally agree (5 points). The combined score of RSCA ranges from 27 to 135, with a higher sum indicates a better resilience level. The Cronbach’s α of RSCA in our study was 0.77. Based on exploratory factor analysis of the designer, the 27 items of RSCA can be grouped into 5 dimensions: goal concentration, emotion regulation, positive perception, family support, and interpersonal assistance.

### Statistical analysis

Descriptive statistics were used to depict general characteristics of LBC. The categorical variables were presented with frequencies and proportions while the continuous variables were described by means and standard deviations (SDs). We dichotomized study subjects into two subgroups based on the presence of any SH behavior collected by MASHS. Univariate logistic regression model with a less strict significance level of *p* < 0.1 was adopted to screen for possible associated factors of SH. Multivariate logistic regression model was latter applied to estimate the adjusted associations between identified factors and SH. Finally, we performed a subgroup analysis only in self-harmed LBC: they were dichotomized by using SH severity (the presence of medium to severe SH) and SH repetition (the presence of more than once SH) separately, associated factors of SH severity and repetition were also analyzed by using univariate and multivariate logistic regression models. All statistical analyses were performed using SPSS 21.0 (SPSS Inc., USA). Statistical significance threshold of *p* value was set at no more than 0.05, two-tailed.

## Results

### General characteristics of participants

By applying inclusion and exclusion criteria mentioned above, altogether 3011 LBC were initially determined. Among them, 28 declined to participate, 10 were latter confirmed over-aged (≥18 years), and 75 were further deleted because of missing information in critical items, which left 2898 subjects for further analysis. The valid response rate was 96.6%. The major characteristics of study subjects were listed in Table 1: the mean age was 14.4 years with a SD of 1.81. Middle school students took the predominant majority (*N* = 1622, 55.97%). A total of 1413 LBC reported engaged in at least one episode of SH, with a lifetime prevalence of 48.80% (95%CI: 47.00–49.70%); slight injury was the commonest type of reported SH (*N* = 1037; 73.39%). In self-harmed LBC, 1017 (71.97%) reported repetitive SH. The overall score of psychological resilience and its SD were 94.42 and 12.85, respectively. Based on the median RSCA score, subjects were dichotomized as low resilience group (RSCA≤94) and high resilience group (RSCA> 94). The average scores of the five specific dimensions of resilience varied, ranging from 3.15 ± 0.78 (emotion regulation) to 3.77 ± 0.73 (goal concentration).

**Table 1:** Descriptive statistics of 2898 LBC analyzed

Variable	*N* (%)	Mean (SD)
Age		14.46 ± 1.81
Gender		
Male	1504 (51.90)	
Female	1394 (48.10)	
Ethnicity		
Han	532 (18.36)	
Zhuang	1624 (56.04)	
Miao	605 (20.88)	
Other	137 (4.72)	
Grade		
Primary school	748 (25.81)	
Middle school	1622 (55.97)	
High school	528 (18.22)	
Education of father		
Illiterate or semi- illiterate	1055 (36.40)	
Primary school	1130 (38.99)	
Middle school or above	512 (17.67)	
Missing	201 (6.94)	
Education of mother		
Illiterate or semi-illiterate	1830 (63.15)	
Primary school	644 (22.22)	
Middle school and above	193 (6.66)	
Missing	231 (7.97)	
Age of father		39.45 ± 5.44
Age of mother		37.78 ± 5.16
Self-harm behavior		
Yes	1413 (48.76)	
No	1485 (51.24)	
Self-harm severity		
Slight	1037(73.39)	
Medium or above	376 (26.61)	
Self-harm repetition		
Yes	1017 (71.97)	
No	396 (28.03)	
Resilience score		94.42 ± 12.85
Goal concentration		3.77 ± 0.73
Interpersonal assistance		3.42 ± 0.85
Emotion regulation		3.15 ± 0.78
Positive perception		3.71 ± 0.71
Family support		3.55 ± 0.68

### Associated factors of SH

As shown in Table 2, variables with *p* values less than 0.10 in univariate analysis were: gender, grade, father’s age, mother’s age, father’s education level, and resilience. After multivariate adjustment, the association between resilience and SH stayed statistically prominent: compared to LBC with lower resilience level (RSCA< 94), a higher resilience level (RSCA≥40) was related to 0.40 folds of SH odds (95%CI: 0.34–0.48). We fitted multivariate logistic regression models which included the 5 specific dimensions of RSCA instead of the combined scores separately, and the results revealed that: except for positive perception, all the other four dimensions were significantly inversely associated with SH. Among the four dimensions, emotion regulation showed the strongest association: every 1 average score increase was associated with an odds ratio (OR) of 0.03 (95%CI: 0.01–0.06) in SH.

**Table 2:** Univariate and multivariate analyses for self-harm behaviors in all left-behind children

Variable	Univariate model		Multivariate model 1		Multivariate model 2	
	OR (95%CI)	OR (95%CI)	*p* value		OR (95%CI)	*p* value
Gender						
Female	Ref					
Male	1.25(1.08–1.44)	< 0.01	0.78 (0.66–0.92)	< 0.01	0.72 (0.61–0.86)	< 0.01
Ethnicity						
Han	Ref					
Zhuang	1.08 (0.89–1.31)	0.45	–	–	–	–
Miao	1.10 (0.87–1.39)	0.44	–	–	–	–
Other	0.87 (0.59–1.26)	0.46	–	–	–	–
Grade						
Primary School	Ref					
Middle School	1.65 (1.39–1.97)	< 0.01	1.66 (1.35–2.04)	< 0.01	1.56 (1.27–1.93)	< 0.01
High school	0.96 (0.76–1.20)	0.70	0.99 (0.77–1.29)	0.96	0.88 (0.67–1.15)	0.36
Father’s age (+ 1 year)	1.02 (1.01–1.03)	0.01	1.02 (0.99–1.04)	0.22	1.02 (0.99–1.05)	0.20
Mother’s age (+ 1 year)	1.02 (1.00–1.03)	0.04	0.10 (0.97–1.03)	0.89	0.10 (0.97–1.02)	0.72
Father’s education level						
Illiterate/semi-illiterate	Ref					
Primary School	0.84 (0.71–1.00)	0.05	0.82 (0.68–0.98)	0.03	0.80 (0.66–0.96)	0.02
Middle School or above	0.77 (0.63–0.96)	0.02	0.80 (0.64–1.01)	0.07	0.78 (0.62–0.99)	0.04
Mother’s education level						
Illiterate/semi-illiterate	Ref					
Primary School	0.91 (0.76–1.09)	0.32	–	–	–	–
Middle School or above	0.87 (0.65–1.17)	0.36	–	–	–	–
Resilience						
RSCA< 94.0	Ref					
RSCA≥94.0	0.42 (0.36–0.48)	< 0.01	0.40 (0.34–0.48)	< 0.01	–	–
Resilience dimensions						
Emotion regulation (+ 1 average score)			–	–	0.03 (0.01–0.06)	< 0.01
Goal concentration (+ 1 average score)			–	–	0.26 (0.10–0.69)	0.01
Interpersonal assistance (+ 1 average score)			–	–	0.17 (0.08–0.36)	< 0.01
Positive perception (+ 1 average score)			–	–	1.83 (0.69–4.84)	0.23
Family support (+ 1 average score)			–	–	0.26 (0.09–0.70)	0.01

Abbreviation: RSCA, the Resilience Scale for Chinese adolescents

### Resilience with SH severity and repetition

**Fig. 1:**
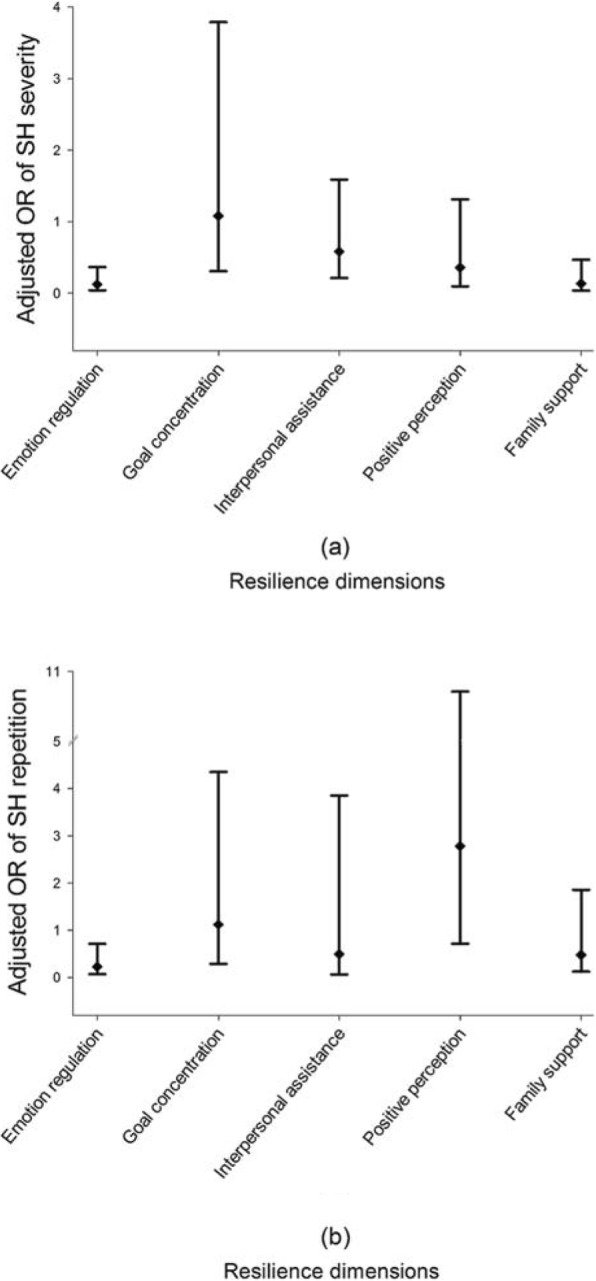
Adjusted odds ratio (OR) with corresponding 95% confidence intervals of SH severity and repetition for every 1 average score increase in dimensions of resilience. **a** Resilience with SH severity: event was defined as medium or above injury, adjusted for gender and grade; **b** Resilience with SH repetition: event was defined as more than once previous SH behaviors, adjusted for gender, grade and mother’s educational level

We further discussed the possible influence of dimensions of resilience on SH severity and repetition in self-harmed LBC. By taking SH severity as the dependent, after adjusted for other possible influencing covariates, only emotion regulation and family support were statistically inversely associated with SH severity: every 1 average score increase of the two dimensions were associated with 0.13 (95%CI: 0.04–0.37) and 0.14 (95%CI: 0.04–0.47) folds of odds in medium or above injuries (Fig. 1, panel a).

By using the same analytical strategy, for 5 dimensions of resilience, only emotion regulation was prominent factor of SH repetition: one unit increase in the average score was related to an OR of 0.23 (95%CI: 0.07–0.71) for repeated SH (Fig. 1, panel b).

## Discussion

In the current study, we thoroughly discussed the association between SH and resilience in a large group of Chinese LBC, an interesting and important topic that has never been adequately addressed. Based on analytical results, we found that, SH is an urgent challenge in LBC, as a hefty 48.8% of self-reported SH prevalence has been revealed, much higher than which in other regions of China [23–25]. Besides, much in accordance with our anticipation, although the strengths and patterns of the associations varied, resilience was in general inversely associated with prevalence, severity, and repetition of SH behaviors.

The positive association between resilience and SH prevalence coincides with prior findings that higher levels of resilience potentially favored SH protection amid Chinese adolescents [26, 27], and non-SH adolescents tend to be more resilient than self-injurers [19]. The adolescent resilience model proposed by Haase [28] reckoned that, by gaining resources from individual, family and society, resilience renders people more prone to obtain resilience-related self-esteem, confidence and life satisfaction. The integrated model of self-injury by Nock [29] emphasized the combined effect of peers, family, schools and society in triggering the advent of self-injury. Therefore, one possible explanation could be that, more resilient individuals enjoy an advantage in multiple resources, which may predispose them to a lowered risk of SH. Additionally, resilience was believed to be a moderator between negative life events and mental health problems [30], and the latter were established risk factors of SH behaviors [31]. With this regard, LBC presented with higher resilience may less likely to be plagued by mental health problems like depression, which in turn, curtails the presence of SH.

Further analysis revealed that, among the five dimensions of resilience, emotion regulation was consistently associated with SH prevalence, severity, and repetition. Among general adolescents, emotion regulation was deemed plays a crucial role in depression and anxiety, as it has been suggested that the unsatisfactory emotion regulation was related to reduced capability in dealing with emotional challenges [32, 33]. In view of the intimate relationship between affective disorders, typically depression and anxiety, and SH behaviors in youngsters [34, 35], one possible explanation could be that, good emotion regulation dissuades self-mutilations engagement. The affect regulation model explains that, self-injury can occur with the purpose of keeping strong negative feelings regulatory [36]. Hence, the lack of emotion regulation ability may relate to susceptibility of immersing in negative mood, and consequently, the adoption of SH behaviors.

Another important finding was that, children with less perceived family support had higher incidence and severer SH behaviors, which was in agreement with several previous studies [37–39]. We found no such association existed in SH repetition, which was discrepant with the findings of Wang et al. [7]. However, different measurements of family support were used. Less intimacy and lower communication frequency between LBC and their migrated parents can increase the likelihood of encountering more loneliness and depression [40, 41]. In LBC, it has been reported that parent-child attachment negatively related to loneliness [40], and longitudinal evidence indicated that this attachment ameliorated the depression of LBC subsequently [42]. Therefore, it is reasonable to hypothesize that family support confers protective effect against SH in LBC by reducing loneliness and depression. Nevertheless, this assumption should be further validated.

Our major findings are of important secular significance. First and foremost, the striking prevalence of SH in LBC highlights the importance and urgency of SH intervention for the government and communities to effectively prevent or reduce such hazardous behavior in this mentally disadvantageous population Besides, the role of psychological resilience should be stressed. This is also a feasible intervention direction, as existing evidence suggests that resilience could be effectively enhanced through various kinds of group-based counseling measures among teens [20, 43].

As aforementioned, when dissected, emotion regulation was the strongest indicator of SH severity and repetition among all 5 dimensions of resilience. One study published in Chinese has already disclosed promising results: intervention on emotion regulation was associated with reduced level of SH frequency in a small group of LBC chosen from a single rural middle school [44]. The approaches the authors adopted, such as the correction of erroneous perceptions on SH, challenging unreasonable faith, and relaxation training, are generally practicable in school or community based resilience intervention programs for LBC.

Moreover, parent-child relationship should also be strengthened. Timely and adequate face-to-face communication is the best way to cultivate intimacy between children and their parents. However, it is unrealistic for LBC. Therefore, other possible substitutes should be considered. Perhaps a higher contact frequency via multiple means of modern communication can be an expedient but ideal choice, such as telephone, instant text message, audio or video chat, etcetera, as some positive conclusions have been reached by empirical studies [45, 46]. Among all available and affordable choices, which communication method outperforms the rest will be an interesting topic to be discussed.

The most important insight of our findings is that, not only the multidimensional nature of psychological resilience should be considered, the characteristics of SH is of identical importance to think about. Intervention measures that focus on different dimensions of resilience in combination of severity and repetition of SH may be the most effective: for LBC engaged in repeated SH, emotion regulation ability building should be stressed, whereas for LBC adopted severer SH behaviors, measures on parent-child relationship improvement could be simultaneously involved.

In this community-based study, we exhaustively discussed the association between resilience and SH with meaningful results. The reliability and validity of our findings can be substantially consolidated by representative sampling strategy and expanded sample size. Nevertheless, several limitations should be noticed. First, this is a cross-sectional study, which means that the causal inference cannot be reached. Second, information bias could exist for all information was collected by self-reporting. Finally, the study subjects were chosen from a single location in west China, thus discretion is needed in extrapolating the status of the whole Chinese LBC population based on our observations. Future studies with representative large sample and prospective design are warranted to further corroborate our findings. Besides, the underlying mechanism behind resilience-SH association should also be investigated.

## Conclusions

SH prevalence is high among Chinese LBC and resilience may serve as a protective factor. Among the five dimensions of resilience, emotion regulation was the strongest associated factor of SH, and it also consistently correlated with SH severity and repetition. Besides, family support played a significant role in SH severity of LBC. These findings highlight the urgency of intervention aims at reinforcing emotion regulation and strengthening parent-child intimacy among this vulnerable population.

## Data Availability

The datasets analyzed during the current study are available from the corresponding author on reasonable request.

## Abbreviations

LBC: left-behind children
MASHS: the Modified version of Adolescents Self-Harm Scale
NLBC: non-left-behind children
RSCA: the Resilience Scale for Chinese Adolescents
SD: standard deviation
SH: Self-harm
SI: suicidal ideation
